# Comparative Transcriptome Profiling Reveals Defense-Related Genes against *Meloidogyne incognita* Invasion in Tobacco

**DOI:** 10.3390/molecules23082081

**Published:** 2018-08-20

**Authors:** Xiaohui Li, Xuexia Xing, Pei Tian, Mingzhen Zhang, Zhaoguang Huo, Ke Zhao, Chao Liu, Duwei Duan, Wenjun He, Tiezhao Yang

**Affiliations:** 1 Department of Tobacco, College of Tobacco, Henan Agricultural University, Zhengzhou 450002, Henan, China; lxh880814@163.com (X.L.); tiantianannsnow@163.com (P.T.); huozhaoguang4555@126.com (Z.H.); zhaoke91@126.com (K.Z.); damengduwoxing@163.com (C.L.); duanduwei1@126.com (D.D.); hwj950221@126.com (W.H.); 2 Nanyang Branch of Henan Province Tobacco Company, Nanyang 473003, Henan, China; xingxuexia2008@163.com; 3 Xiaogan Agricultural Technical Extension Station, Xiaogan 432000, Hubei, China; yczhangmingzhen@163.com

**Keywords:** tobacco, *Meloidogyne incognita*, transcriptome profiling, resistance

## Abstract

Root-knot nematodes *Meloidogyne incognita* are one of the most destructive pathogens, causing severe losses to tobacco productivity and quality. However, the underlying resistance mechanism of tobacco to *M. incognita* is not clear. In this study, two tobacco genotypes, K326 and Changbohuang, which are resistant and susceptible to *M. incognita*, respectively, were used for RNA-sequencing analysis. An average of 35 million clean reads were obtained. Compared with their expression levels in non-infected plants of the same genotype, 4354 and 545 differentially expressed genes (DEGs) were detected in the resistant and susceptible genotype, respectively, after *M. incognita* invasion. Overall, 291 DEGs, involved in diverse biological processes, were common between the two genotypes. Genes encoding toxic compound synthesis, cell wall modification, reactive oxygen species and the oxidative burst, salicylic acid signal transduction, and production of some other metabolites were putatively associated with tobacco resistance to *M. incognita*. In particular, the complex resistance response needed to overcome *M. incognita* invasion may be regulated by several transcription factors, such as the ethylene response factor, MYB, basic helix–loop–helix transcription factor, and indole acetic acid–leucine-resistant transcription factor. These results may aid in the identification of potential genes of resistance to *M. incognita* for tobacco cultivar improvement.

## 1. Introduction

Root-knot nematodes (RKNs) *Meloidogyne incognita* are one of the most destructive pathogens, limiting plant productivity and quality. At present, *M. incognita* is controlled in crops by using chemical nematicides, adequate cultural practices, and soil fumigants. However, using cultivars with resistance or tolerance genes has proved to be the most effective and environmentally friendly strategy for managing *M. incognita* [1]. Some predominant resistance genes have been identified and mapped on the chromosomes of host plants, e.g., the *Mae* and *Mag* genes in peanut [2], *Mi* genes in tomato [3], and *Me3* genes in pepper [4]. However, RKNs have more than 3000 potential host plants, including tobacco [5]. Tobacco (*Nicotianatabacum* L.) is not only a model plant for molecular research but also an important economic crop in approximately 120 countries worldwide [6]. However, the yield and mortality of susceptible plants are remarkably reduced with increased *M. incognita* infection. The resistance to *M. incognita* varies considerably among tobacco varieties, and exploring the underlying molecular mechanisms of resistance to *M. incognita* may facilitate the breeding efforts for enhanced-resistance genotypes. Plants have evolved various basal and inducible mechanisms to withstand the invasion by nematodes. Generally, the mechanisms underlying the resistance response in plants involve cell wall modification, proteins with antimicrobial activities, and secondary metabolites, e.g., phytoanticipins and phytoalexins [7]. The plant cell wall is composed of hemicelluloses, cellulose microfibrils, and pectin and represents a physical barrier to infection during attack by pathogens. Nematodes have evolved complex mechanisms to break down the barrier; in particular, they secrete a series of cell wall-modifying enzymes into the plant root [8]. Once nematodes successfully penetrate into root tips of host plants, resistant genotypes can recognize microbe-associated molecular patterns using cell surface-localized pattern recognition receptors. Subsequently, plants involve resistance genes to recognize specific effectors and trigger effector-triggered immunity; finally, systemic acquired resistance (SAR) is established. SAR and the expression of pathogenesis-related genes (PRs) are triggered by salicylic acid (SA), which plays a crucial role in decreasing the susceptibility of plants to nematode infections [9]. 

Resistance to RKNs, mediated by hypersensitivity reaction, is characterized by the production of reactive oxygen species (ROS), overaccumulation of which—also known as oxidative burst—usually causes cells to constantly live in an oxidizing environment, eventually leading to cell death [10]. To overcome the adverse effects of the ROS burst, RKNs produce a series of peroxiredoxins, such as peroxidases (PODs), thioredoxins, catalases, and superoxide dismutase. Peroxiredoxins, which are localized in tissues surrounding the cuticle of second-stage juveniles (J2s) and in the hypodermis, in close contact with plant cells, play a crucial role in scavenging host-derived ROS. Numerous genes are activated or modulated in plant hormone signaling pathways involving SA and jasmonic acid (JA) in response to nematode infection via the modulation of different transcription factors (TFs). In particular, the application of exogenous SA and JA has been reported to induce RKN resistance responses in tomato [11,12]. Currently, little is known regarding the precise role of hormones and TFs in nematode infestation, owing to complex interactions between various signaling pathways. Thus, identification of defense response networks among host plants and RKNs is still needed.

Comparative transcriptome analysis using RNA sequencing (RNA-Seq) has been widely used to investigate non-model and model species [13]. Unlike traditional microarray analysis, RNA-Seq has significant advantages, e.g., large amounts of accurate expression data, broader capability of dynamic range detection, high sensitivity and reproducibility of replicates, exploration of novel genes, as well as the precise location of splice isoforms [14,15]. This approach has been successfully applied in studying plant–nematode interactions during *M. incognita* infestation in *Phaseolusvulgaris* [16], *Cucumismetuliferus* [17], and *Solanumlycopersicum* [18]. However, available information concerning interactions between tobacco and *M. incognita* is limited, in contrast to recent advances in other plants. As *M. incognita* invasion is initially sensed by tobacco roots; the root is the ideal tissue for determining the tobacco resistance response and adaptation to *M. incognita*.

In this study, tobacco was used as a model to identify the physiological and transcriptional changes in the resistant K326 and susceptible Changbohuang genotype in response to *M. incognita* invasion. This study aimed to identify relevant genes and pathways associated with tobacco resistance and elucidate the complex regulatory networks associated with the response to *M. incognita*. 

## 2. Results

### 2.1. Effects of Nematode Infection on the Phenotypic Traits of Different Tobacco Genotypes

**Figure 1:**
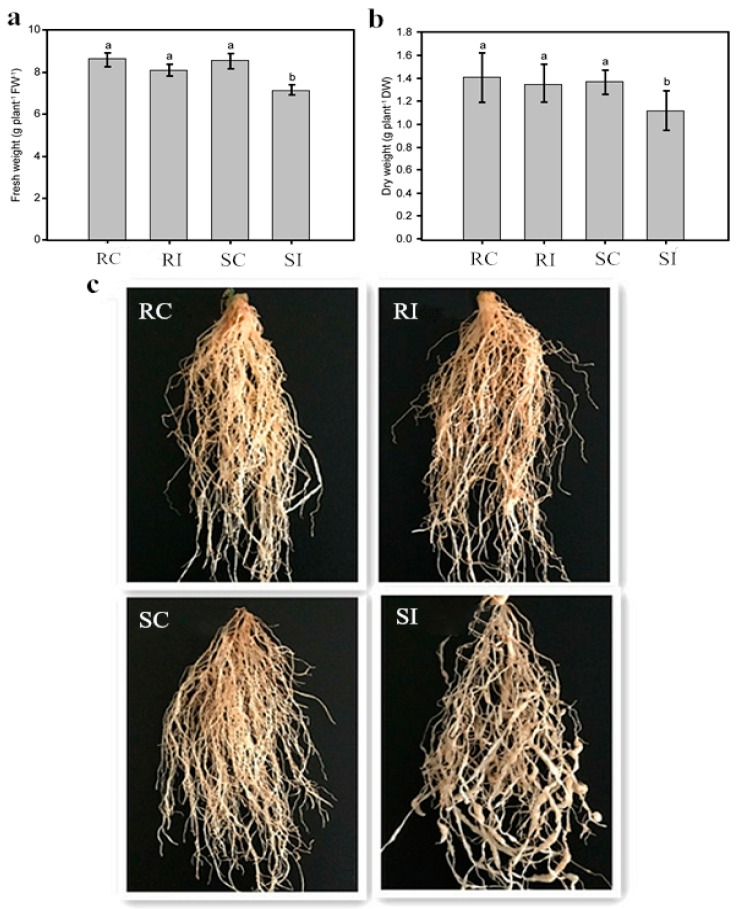
Phenotypic traits of K326 and Changbohuang. (**a**) Fresh weight. (**b**) Dry weight. (**c**) Symptoms of nematode infection. Notes: Means followed by the small letters are significantly different at 0.05 levels, respectively.

Two typical tobacco genotypes: non-inoculated K326 (resistant control, RC), inoculated K326 (resistant inoculated, RI), Changbohuang (susceptible control, SC), and Changbohuang (susceptible inoculated, SI) were used in this study. The results revealed significant difference in the biomass of nematode-inoculated and control plants. Line K326 was only slightly affected in comparison with Changbohuang (Figure 1). The relative root fresh weight (treated/control) was 97% for K326 and 84% for Changbohuang (Figure 1a) and the relative root dry weight was 96% for K326 and 82% for Changbohuang (Figure 1b). Figure 1c shows that the nematodes penetrated the roots of Changbohuang, and then transformed the injured root cells into giant cells or “knots”. These deformities severely impeded water and nutrient absorption in Changbohuang, whereas the resistant cultivar K326 continued to grow and develop normally. 

### 2.2. Resistance and Susceptibility of the Two Genotypes

The disease index (DI) was investigated in plants after 60 days of infection with RKNs, as described by Wang et al. [19]. The average DIs of 19.1 and 86.7 were obtained for K326 and Changbohuang, respectively. According to the DI scale [19], reactions to RKNs are categorized as follows: DI < 20 = resistant; DI 25.1–50.0 = moderately resistant; DI 50.1–75.0 = moderately susceptible; and DI > 75.1 = susceptible. In a previous study, different commercial varieties of tobacco have been classified using this scale [19]. Hence, K326 was considered resistant and Changbohuang was considered susceptible, which corroborated the findings of previous studies [19,20].

### 2.3. Determination of Physiological Changes in Response to M. incognita

**Figure 2:**
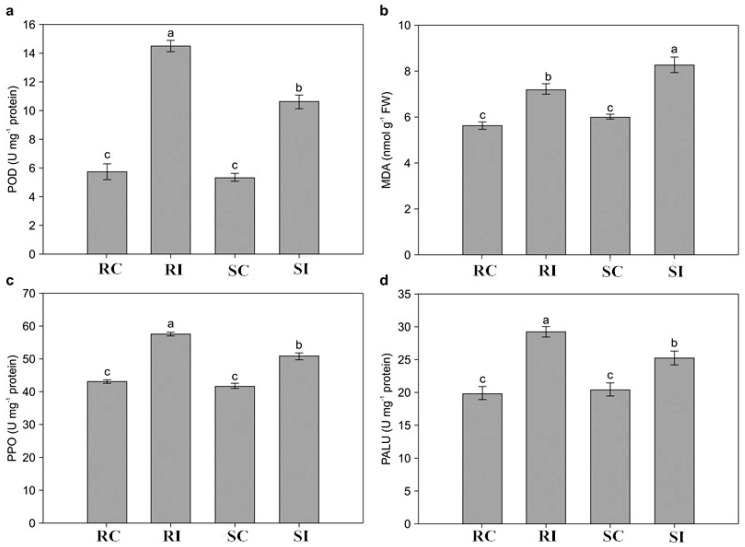
Changes in physiological parameters in the resistant (R) and susceptible (S) genotype in response to *Meloidogyne incognita* infection. Three independent experimental replicates were analyzed for each sample, and the data are presented as the mean ± SE (*n* = 3). Different lowercase letters represent significant differences (*p* < 0.05). POD = peroxidase; PPO = polyphenol oxidase; PAL = phenylalanine ammonialyase; MDA = malondialdehyde.

To evaluate physiological and biochemical changes caused by *M. incognita* invasion, we measured activities of POD, polyphenol oxidase (PPO), and phenylalanine ammonia lyase (PAL), as well as the content of malondialdehyde (MDA). No considerable differences in the four parameters were noted between RC and SC before *M. incognita* infection. However, the values remarkably increased in RI and SI samples after *M. incognita* infection (Figure 1). The levels of POD, PPO, and PAL activities were significantly higher in RI than in SI samples, whereas the MDA concentration remarkably decreased in the RI samples compared with that in the SI samples (Figure 2).

### 2.4. Histopathological Response to Nematode Infection

**Figure 3:**
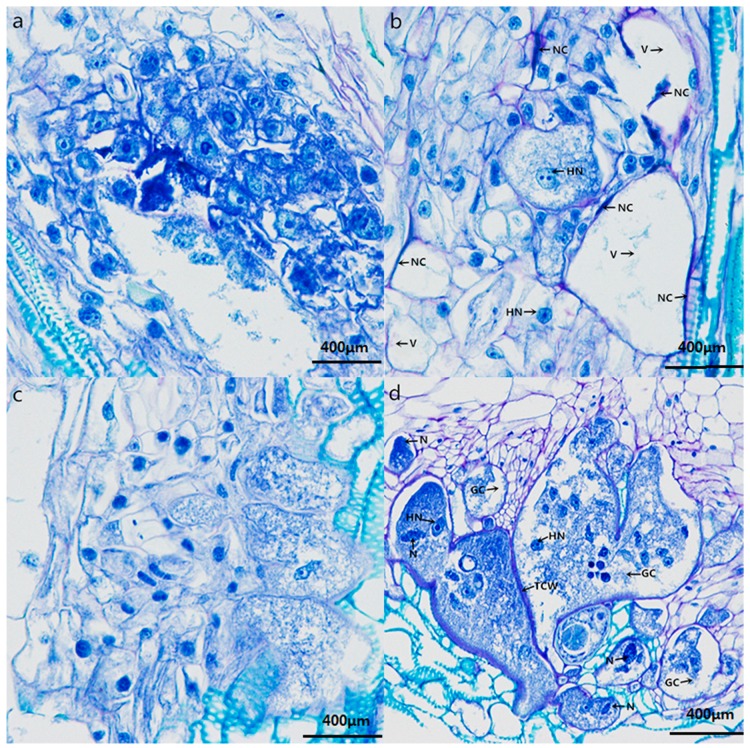
Histopathological observation between resistant and susceptible tobacco cultivars inoculated with nematodes. (**a**,**b**) are RC and RI root sections. (**c**,**d**) are SC and SI root sections. GC, giant cell; N, nematode; NC, necrosis cell; V, vacuole; HN, hypertrophied nucleus; TCW, thickened cell wall.

Nematode juveniles penetrated the roots and established feeding sites known as giant cells in both resistant K326 and susceptible Changbohuang. As shown in Figure 3, the first obvious difference between the two genotypes was observed at 7 d post-inoculation, when the giant cells adjacent to the nematode-invaded cells in the resistant roots showed hypersensitive necrosis (Figure 3b), whereas, no necrosis was observed in the susceptible roots (Figure 3d). Some large vacuoles were observed in the giant cells developed in the resistant roots (Figure 3b), whereas, the giant cells showed fewer vacuoles, and variable number of nucleoli and hypertrophied nuclei in the susceptible roots (Figure 3d). Giant cell complexes with thick cell walls and dense cytoplasm were constantly enlarged in the susceptible roots (Figure 3d), but most of the giant cells appeared to be on the verge of collapsing as they were devoid of cytoplasm, and the cell walls between giant cells were thin in the resistant roots (Figure 3b). 

### 2.5. Transcriptional Changes in Tobacco in Response to M. incognita

Since nematode invasion is initially sensed by tobacco roots, roots are the ideal tissue for investigating the underlying mechanism of resistance to plant disease caused by *M. incognita*. According to the method described by Postnikova et al. [21], roots of the resistant and susceptible tobacco genotype were collected 7 days after inoculation with RKNs, thoroughly rinsed with distilled water, and used for RNA-Seq of four cDNA libraries, RC, RI, SC, and SI. Approximately 48 million raw reads were generated (Table 1). After low-quality reads were filtered out, more than 35 million clean reads were obtained for each sample (Table 1). Approximately 82% of the clean reads were mapped to the reference genome, of which 1% were mapped to multiple locations and 81% were mapped to unique locations (Table 1).

**Figure 4:**
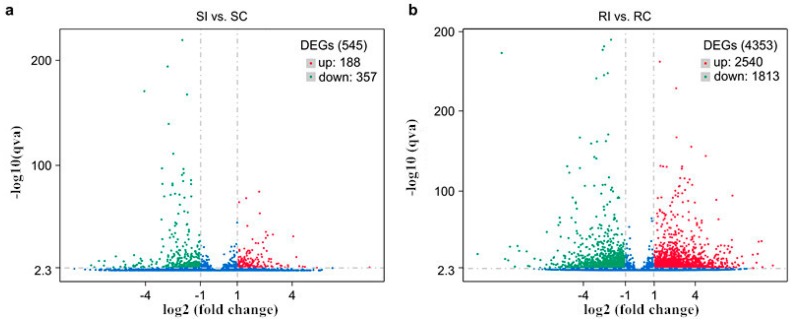
Volcano plots of differential gene expression. The log2 (inoculated/non-inoculated) indicates the expression level for each gene. Green dots represent downregulated genes, and red dots represent upregulated ones. Blue dots represent genes that showed no differential expression. (**a**) Susceptible genotype, inoculated vs. non-inoculated (SI vs. SC). (**b**) Resistant genotype, inoculated vs. non-inoculated (RI vs. RC).

**Figure 5:**
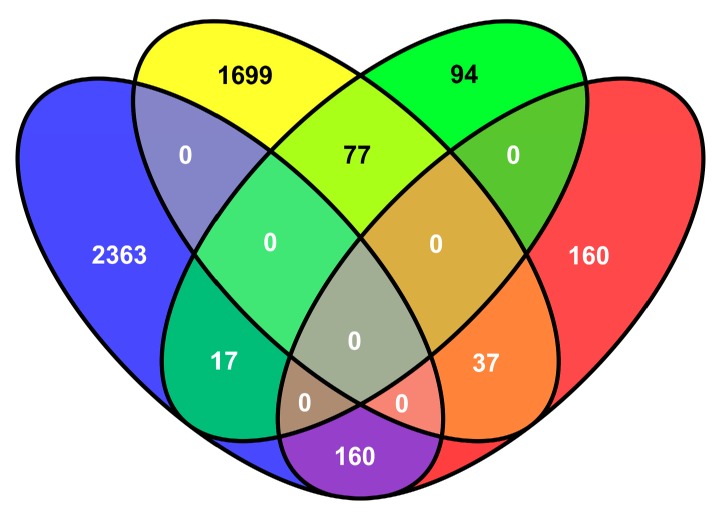
Venn diagram of differential gene expression. Blue and yellow ovals represent upregulated and downregulated differentially expressed genes (DEGs) in the resistant genotype, respectively. Green and red ovals represent upregulated and downregulated DEGs in the susceptible genotype, respectively.

The genes differentially expressed in the resistant and susceptible tobacco genotype in response to *M. incognita* infection were compared using|log2 (fold change)| ≥ 1 and *p* < 0.05 as significant cutoff criteria. By using these criteria, we detected 4353 differentially expressed genes (DEGs) in the resistant genotype, of which 2540 were upregulated and 1813 were downregulated. We also detected 545 DEGs in the susceptible genotype, of which 188 were upregulated and 357 were downregulated (Figure 4). Despite the different genetic backgrounds, 291 DEGs were common for both genotypes (Figure 5) and were putatively considered to be associated with tobacco resistance to *M. incognita*. Among these genes, 37 upregulated and 17 downregulated DEGs were shared by the two genotypes (Figure 5), indicating some common pathways involved in the response to *M. incognita* inoculation. Furthermore, 160 DEGs were upregulated in K326 and downregulated in Changbohuang (Figure 5). Moreover, 77 of the downregulated DEGs in K326 were upregulated in Changbohuang (Figure 5).

### 2.6. Gene Ontology and Kyoto Encyclopedia of Genes and Genomes Enrichment Analyses of Resistance-Related DEGs

**Figure 6:**
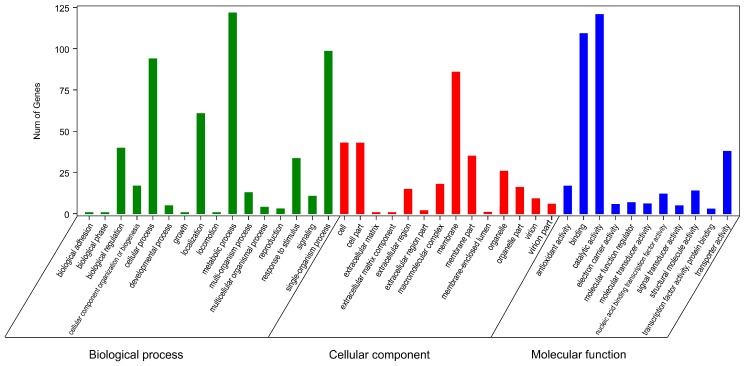
Gene ontology analysis revealed significant enrichment of the 291 common differentially expressed genes in certain categories.

The Gene Ontology (GO) and Kyoto Encyclopedia of Genes and Genomes (KEGG) enrichment analyses revealed the biological processes, cellular components, molecular functions, and metabolic pathways associated with the identified transcripts from tobacco roots. The 291 putative resistance-related DEGs were divided into three principal GO categories: biological process, cellular component, and molecular function (Figure 6). Sixteen significant terms were grouped into biological processes, such as signaling, response to stimulus, and biological regulation. Membrane represented the largest proportion in the cellular component category. Molecular function included 11 significantly enriched terms, among which TF, signal transducer, transporter, and antioxidant activity are normally regarded to be related to plant resistance (Figure 6). 

KEGG enrichment analysis was conducted to evaluate nematode resistance-related pathways. A total of 36 pathways were enriched (Supplementary Material Table S2). Among them, six pathways were significantly enriched (*p*-value < 0.05), including “phenylpropanoid biosynthesis”, “tropane, piperidine, and pyridine alkaloid biosynthesis”, “tyrosine metabolism”, “isoquinoline alkaloid biosynthesis”, “pentose and glucuronate interconversions”, and “starch and sucrose metabolism” (Supplementary Material Table S2). These data suggest that some secondary metabolites might participate in the resistance to nematodes in tobacco. 

### 2.7. Antioxidant Enzymes

**Figure 7:**
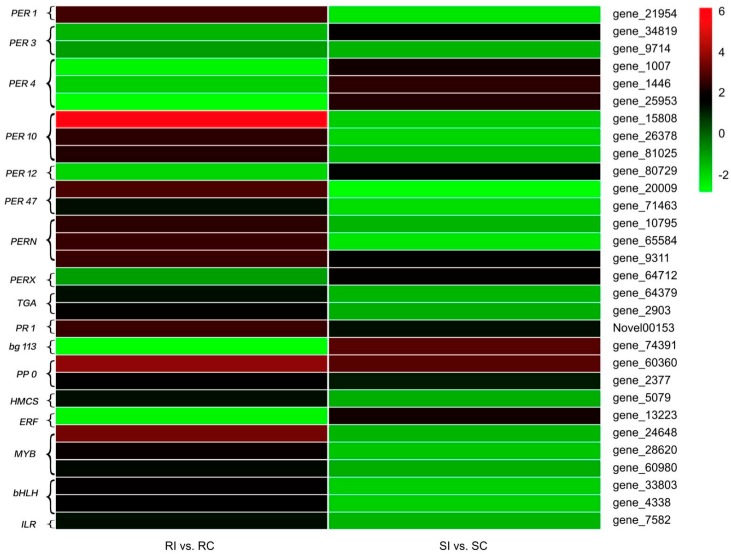
Comparison of the resistance-related genes expressed in the resistant (R) and susceptible (S) genotype in response to *Meloidogyne incognita* infection using heatmaps. The colored bars represent the log_2_ fold change in the RI vs. RC and SI vs. SC libraries.

Peroxidases (PRXs) are known to be the key antioxidant enzymes that regulate plant extracellular H_2_O_2_ levels [22]. In this study, 16 PRX-encoding genes were identified (Figure 7). Among these, one *PER1*, three *PER10*, two *PER47*, two *PERN*, and one *PERN1* gene were upregulated in the resistant genotype but downregulated in the susceptible genotype after *M. incognita* infection. However, one *PER3*, three *PER4*, one *PER12*, and one *PERX* gene exhibited the opposite trend in the two genotypes. In addition, one gene encoding *PER3* was repressed in both the resistant and susceptible genotypes (Figure 7).

### 2.8. Signal Transduction

SA has been reported to act as a signal molecule, mediating a complex signaling network in plant defense reactions [23]. Three genes related to SA signal transduction were detected in our study. Two TGA-encoding genes were activated in the resistant genotype but repressed in the susceptible one. However, the expression of *PR1* was upregulated, with 2.5594- and 1.2235-fold increases in the resistant and susceptible genotype, respectively (Figure 7). 

### 2.9. Secondary Metabolism

Secondary metabolism, such as the biosynthesis of phenylpropanoids, alkaloids, and terpenoids, has been shown to be associated with the plant defense system via cell wall enforcement and phytoalexin synthesis [24]. One gene, encoding beta-glucosidase 13 in the phenylpropanoid biosynthesis pathway, was downregulated, with a 2.897-fold decrease, in the resistant genotype but upregulated, with a slight, 2.937-fold increase in the susceptible genotype (Figure 7). 

After *M. incognita* infection, two genes encoding PPO, mapped to the alkaloid biosynthesis pathway, were both activated in the resistant and susceptible genotype (Figure 7). 

Among the common DEGs, one gene, encoding hydroxymethylglutaryl-coenzyme A synthase (HMCS), which was upregulated in the resistant genotype but repressed in the susceptible genotype (Figure 7), was identified in the terpenoid backbone biosynthesis pathway.

### 2.10. Transcription Factors

TFs have been proposed to play important roles in the regulation of gene responses to biotic and abiotic stresses [25,26]. The methods of TF classification and identification are provided in the Plant Transcription Factor Database (PlnTFDB; http://plntfdb.bio.uni-potsdam.de/v3.0/). A total of seven DEGs related to nematode resistance in tobacco have been annotated and classified into four TF families (ethylene response factor (ERF), myoglobin (MYB), basic helix-loop-helix (bHLH), and indole acetic acid–leucine-resistant (ILR); Figure 7). The ERF, MYB, and bHLH families are known to be responsible for pathogen challenges [24]; however, the involvement of the ILR family has not previously been reported. In our study, most of the TF families were activated in the resistant genotype and repressed in the susceptible one, such as the MYB, bHLH, and ILR families; in particular, the expression of the *MYB* gene (gene_24648) in the RI samples was 10-fold higher than that in the RC samples (Figure 7). However, the ERF family was repressed in the resistant genotype and activated in the susceptible one (Figure 7). These differentially expressed TF families may cause downstream physiological alterations in host plants to adapt to *M. incognita* invasion.

### 2.11. Verification of RNA-Seq Data by Quantitative Real-Time Polymerase Chain Reaction

**Figure 8:**
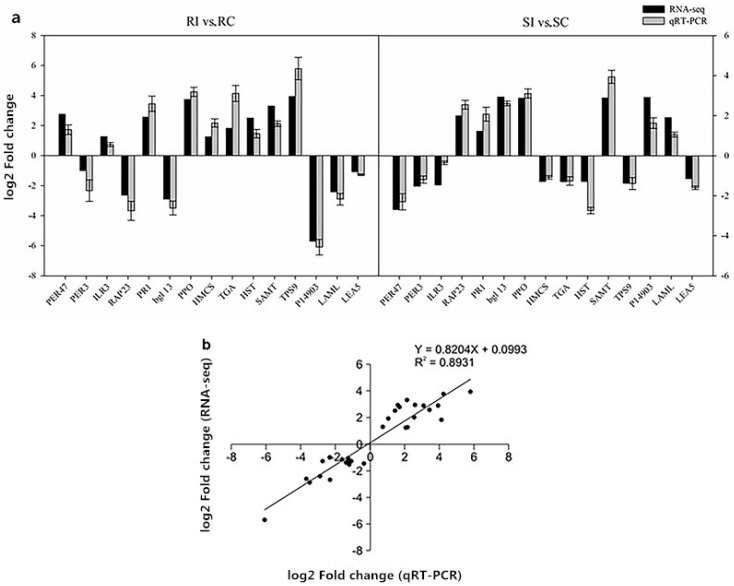
(**a**) Quantitative real-time polymerase chain reaction (qRT-PCR) validation of selected differentially expressed genes. (**b**) The RNA-Seq data (X-axis) were plotted against the qRT-PCR data (Y-axis). The function of the regression line and the *R*^2^ value are shown.

The accuracy and reproducibility of the RNA-Seq data were validated by selecting 15 putative nematode resistance-related genes in the K326 and Changbohuang genotypes for quantitative real-time polymerase chain reaction (qRT-PCR) analysis (Supplementary Material Table S1). As expected, these candidate genes had similar expression tendencies, and the correlation coefficient between the RNA-Seq and qRT-PCR data was 0.8931 (Figure 8). Thus, this result suggested that our RNA-Seq data were accurate and reliable.

## 3. Discussion

Cost-effective global transcriptome analyses, conducted to reveal comprehensive resistance mechanisms underlying host–nematode interactions, have been reported in model [27] and non-model plants [28,29]. However, no similar study has been conducted in tobacco as a model plant, which is widely used in fundamental biological process-related and molecular studies. In our study, K326 and Changbohuang, which showed significantly different DIs and physiological responses (Figure 2), were selected for transcriptome sequencing as *M. incognita*-resistant and susceptible genotypes, respectively. An average of 35 million clean reads were obtained, and approximately 82% of the sequences were successfully mapped to the reference genome (Table 1), which may allow an expansion of the publicly available transcriptome pool for tobacco. Despite the different genetic backgrounds of the plants, 291 DEGs putatively relevant to resistance to *M. incognita* were selectively analyzed, which may provide an insight into the complex resistance mechanism in tobacco. GO and KEGG analyses clearly implicated several pathways, including those associated with signal transduction, antioxidant activity, TFs, and some secondary metabolites, which may provide additional candidate genes for understanding the defense process (Figure 6 and Supplementary Material Table S2).

Phytoalexins and cell wall lignins act as chemical and physical plant barriers, respectively, to resist pathogen attacks [30]. PAL is the key enzyme of phenylpropanoid metabolism and is closely related to the production of phenolic, coumaric, and caffeic compounds as toxic chemical substances during nematode infection. In addition, phenolic compounds are catalyzed by PPO into anthraquinones or terpenoids, which can inhibit the invasion of pests and diseases. POD is responsible for multiple crosslinking of cell wall lignins, and lignins act as a potential physical barrier against nematodes [31]. Our investigation suggested that the induction of PAL, PPO, and POD activities after *M. incognita* inoculation was significantly higher in the resistant genotype than in the susceptible one (Figure 2), consistent with the observation regarding the interrelation between *C. metuliferus* and *M. incognita* [17]. Furthermore, a similar tendency was observed in the expression of the genes encoding PPO and HMCS, which was validated using qRT-PCR (Figure 7). 

Changes in the accumulation of cell wall-modifying proteins (CWMPs) suggest that they play a crucial role in nematode–plant interactions [32]. Hewezi et al. [33] showed that the establishment of nematode-induced feeding structures was mediated by the coordinated expression of CWMPs. In this study, suppression of the CWMP beta-glucosidase13 (Figure 7) in the resistant genotype may suggest that the degradation of the cell wall component cellulose was inhibited, resulting in the hardening and strengthening of the cell wall to form a physical barrier, protecting the plant from nematode penetration. Thus, the main differences between the resistant and susceptible genotype were that the former could be more efficient in forming barriers against *M. incognita* infection.

ROS production and the oxidative burst are induced in plant cells under pathogen attacks [34]. To avoid oxidative stress and damage to cellular functions, plants express scavenging enzymes such as superoxide dismutase, catalase, and POD, which act as the antioxidant defense system [35]. In our study, POD was remarkably increased in RI and SI samples compared with its levels in RC and SC samples, respectively, but was remarkably higher in SI than in RI (Figure 2), indicating that ROS scavenging mainly depended on POD activity after *M. incognita* infection. Furthermore, the expression of 16 PRX genes varied markedly between the two genotypes (Figure 7), implying that these genes might participate in complex regulation in tobacco in response to *M. incognita* invasion. These findings are consistent with those of previous studies on *M. incognita*-inoculated plants, such as cowpea [36]. MDA was maintained at a higher level in the resistant genotype than in the susceptible one (Figure 2), indicating that the former had a stronger ability to maintain an intracellular ROS balance. Thus, antioxidants may be involved in early stages of the defense response in tobacco. 

The results of the histopathological observation revealed that different genotypes may be responsible for different results. Nematodes that penetrated the roots of K326 were poorly developed, suggesting that K326 is a poor host for nematodes and that it could not supply enough nutrients for the nematodes to complete their life cycle. This result is consistent with the findings of a previous study [37]. The results of the present study indicate that the resistance of K326 to nematodes is strongly associated with hypersensitive reaction (HR), which is similar to the resistance mechanism of coffee and tomato to nematodes [38,39]. Another interesting phenomenon observed in the infected K326 was the occurrence of large vacuoles in the giant cells. It is supposed that the large vacuoles in the resistant roots of K326 were filled with toxins and hydrolases that deprived the nematodes of nutrients and led to the collapse of giant cells, whereas, in the susceptible roots of Changbohuang, nematode feeding did not cause the formation of large vacuoles. Resistance mediated by HR seems to be mainly associated with nematode feeding site degeneration [40].

Perception of pathogen invasion elicits a defense response to the pathogen, such as the activation of SA, a vital signaling molecule, followed by triggering of SAR and activation of PRs [41]. In the present study, two *TGA* genes, responsible for SA biosynthesis and signal transduction, were upregulated in the resistant genotype, and one *PR1* gene was upregulated in the susceptible genotype after *M. incognita* invasion (Figure 7), which was consistent with the findings of previous studies on the role of PRs in mediating plant resistance to pathogens [42]. Molinari et al. [43] showed that SAR was caused by nematode infection in tomato. Similar responses may occur in tobacco in response to *M. incognita* invasion; however, this hypothesis needs to be tested in future studies.

Our transcriptome analysis suggested that numerous genes were up- and downregulated during the interaction of *M. incognita* with tobacco. The massive regulatory network associated with *M. incognita* invasion was mediated by a large-scale and complex transcriptional regulation involving many TFs, which are sequence-specific DNA-binding factors able to activate or repress downstream target genes [44]. Some core sets of these TFs, including WRKY, ERF, bHLH, NAC, and MYB families, have been reported to be differentially expressed in response to biotic stresses [24], whereas others, such as the ILR family, have not been reported to be associated with disease resistance. Among these, members of the *ERF*, *MYB*, *bHLH*, and *ILR* families were identified to be involved in the response to *M. incognita* invasion, based on their differential expression levels, in this study (Figure 7). Most of these TFs were remarkably upregulated in the resistant genotype (Figure 7). Nonetheless, to understand specific mechanisms of action of these TFs in the resistance of tobacco to *M. incognita*, changes in their expression need to be further studied to reveal their crucial functions in the modulation of defense processes in tobacco.

In conclusion, physiological indexes and transcriptomic analysis were used to investigate the defense mechanism-related expression profiles in two tobacco genotypes in response to *M. incognita* invasion. Numerous DEGs were detected in K326 and Changbohuang in response to *M. incognita* invasion, potentially resulting in toxic compound synthesis, lignin deposition, and cell wall modification, ROS generation and the oxidative burst, SA signal transduction, and defense-related protein expression. In particular, the activation of several TFs, such as ERF, MYB, bHLH, and ILR, implies their involvement in the regulation of the complex resistance response induced to overcome *M. incognita* infection at the transcriptional level. Taken together, these results may facilitate our understanding of the molecular mechanisms induced in tobacco by *M. incognita* invasion and can form the basis for the identification of potential genes of resistance to nematodes, as well as aid in the construction of a genetic map for tobacco.

## 4. Materials and Methods

### 4.1. Preparation of Tobacco Material and Nematode Inoculation

*M. incognita* nematodes were propagated on susceptible tomato plants (*S. lycopersicum* L. “Rutgers”) in a greenhouse. Egg masses were isolated from triturated tomato roots by using 0.5% NaOCl and then hatched at 26 °C. Freshly hatched J2s were preserved in deionized water. The experiment was conducted in a growth chamber under controlled conditions (16/8-h light/dark cycle at 26 °C and photosynthetic photon flux density of 350 μmol·m^−2^·s^−1^). The roots of 15 seedlings at a 10-leaf stage were inoculated with approximately 1000 J2s in 5 mL of deionized water, while control seedlings were mock-inoculated with the same volume of deionized water. A randomized complete block design was used in this experiment with three replicates.

### 4.2. Determination of Physiological Parameters and Resistance to M. incognita

Root samples were harvested at 7 days post-inoculation from both *M. incognita*- and mock-inoculated plants of K326 and Changbohuang. Samples were collected from three plants each. The POD activity was measured as described by Rao et al. [45]; PPO and PAL were assayed according to Yingsanga et al. [46]. The MDA content was measured using the method of Zhou and Leul [47]. At 60 days, DIs were determined to calculate the resistance to *M. incognita*, as described by Wang et al. [19].

### 4.3. Histological Experiments

Histopathological observation studies were conducted to determine the root cellular response in resistant and susceptible tobacco. The root tips were harvested from two genotypes at 7 d post inoculation. The roots were dehydrated with a graded ethanol series (40%, 70%, 85%, 90%, and 100%) and embedded in 58 °C-melting-point paraffin. Serial sections of thickness 10 μm were cut using a rotary microtome (Leica Microsystems, Shanghai, China), and then deparaffinized and hydrated with water (Xylene I-20 min, Xylene II-20 min, 100% alcohol I-5 min, 100% alcohol II-5 min, and 75% alcohol-5 min). The sections were stained for 2–5 min in toluidine solution, washed with water, observed under a microscope (Nikon, Tokyo, Japan), differentiated based on color, and dried in an oven [48].

### 4.4. Illumina Sequencing Andreads Mapping to the Reference Genome

The roots were thoroughly rinsed in tap water at 7 days post-inoculation and then immediately frozen in liquid nitrogen until RNA extraction. The treatments were as follows: non-inoculated K326 (RC), inoculated K326 (RI), non-inoculated Changbohuang (SC), and inoculated Changbohuang (SI). Total root RNA was extracted using the TRIzol reagent (Invitrogen, Carlsbad, CA, USA), and then four sequencing libraries were constructed using the NEBNext Ultra directional RNA library prep kit for Illumina (New England Biolabs, Ipswich, MA, USA), in accordance with the manufacturer’s protocols. After cluster generation, the library preparations were sequenced on an Illumina HiSeq 2000 platform, and 150-bp paired-end reads were generated.

Raw reads in the FASTQ format were processed using Perl scripts. In this step, clean reads were acquired by removing reads containing an adapter, low-quality reads, and reads containing poly N. At the same time, Q20, Q30, and the GC content were calculated for the clean data. All subsequent analyses were based on high-quality clean reads. The reference genome and gene annotation files were downloaded directly from the genome website. An index of the reference genome was built using Bowtie v2.2.3 (https://sourceforge.net/projects/bowtie-bio/files/bowtie2/2.3.0/), and clean reads were aligned to the reference genome (ftb://anonymous@ftp.solgenomics.net/genomics/Nicotiana_tabacum/assembly/K326) using TopHat v2.0.12 (http://tophat.cbcb.umd.edu/). We selected TopHat as the mapping tool because it can generate a database of splice junctions based on the gene model annotation file and thus produces better mapping results than other, non-splice mapping tools do.

### 4.5. Gene Annotation and Data Analysis

Clean reads were aligned to the K326 tobacco genome using the TopHat v2.0.12 software. A fragments per kilobase per million (FPKM) analysis, which simultaneously considers the sequencing depth and length for this count [49], was used to measure the gene expression levels. Genes with an expression level of at least 1 FPKM in at least one sample were retained after removing genes with low expression levels. The DEGSeq v1.20 (http://bioinfo.au.tsinghua.edu.cn/software/degseq) was used to analyze the differential expression between two samples. The false discovery rate was controlled by adjusting the *p*-values by using the Benjamini and Hochberg method [50]. Thresholds of an adjusted *p*-value of < 0.05 and the absolute value of the log2 ratio of ≥1 were set to evaluate the significance of DEGs. GO annotation of DEGs was performed using the GO seq v2.12 (http://bioconductor.org/packages/goseq/). Next, the KEGG enrichment analysis was implemented using the KOBAS software v2.0 (Center for Bioinformatics, Peking University, Beijing, China). 

### 4.6. Validation of RNA-Seq Data by qRT-PCR

Fifteen DEGs were selected to test the reliability of the RNA-Seq data by qRT-PCR with three biological replicates. Gene-specific primers were designed using Primer Premier 5.0 (see Supplementary Material Table S1) and synthesized by Invitrogen Trading Co., Ltd. (Shanghai, China). Total RNA extraction, cDNA synthesis, qRT-PCR, and statistical analyses were performed as previously described [51]. Amplification was conducted using SYBR Premix Ex Taq™ (Toyobo Co., Ltd., Osaka, Japan). The RT-PCR program was as follows: initial denaturationat 95 °C for 5 min, followed by 40 cycles of denaturation at 95 °C for 30 s, annealing at 54 °C for 30 s, and extension at 72 °C for 30 s and a final extension at 72 °C for 10 min. Relative quantification of gene expression levels was performed using Actin 11 as an internal reference gene for normalization. The comparative threshold cycle (Ct) 2^−ΔΔCt^ method was used to calculate fold changes in gene expression [52].

## Figures and Tables

**Table 1:** Statistical analysis of transcriptome data in the resistant (R) and susceptible (S) genotype.

Treatment	Raw Reads	Clean Reads	Total Mapped Reads (%)	Reads Mapped to Multiple Locations (%)	Uniquely Mapped Reads (%)
RC	56,013,982	53,593,540	45,054,720 (84.07%)	732,943 (1.37%)	44,321,777 (82.7%)
RI	46,922,130	45,634,502	37,457,092 (82.08%)	676,444 (1.48%)	36,780,648 (80.6%)
SC	44,485,484	43,237,234	35,906,665 (83.05%)	654,003 (1.51%)	35,252,662 (81.53%)
SI	47,795,944	46,339,310	38,154,998 (82.34%)	701,137 (1.51%)	37,453,861 (80.83%)
Average	48,804,385	47,201,146	39,143,369 (82.89%)	691,132 (1.47%)	38,627,522 (81.42%)

## Supplementary Materials

The following supplementary materials are available online: Table S1: Validated list of gene primers used for quantitative real-time PCR and Table S2: Kyoto Encyclopedia of Genes and Genomes enriched pathways.
