# Supplementary material for: Comparative Transcriptome Profiling Reveals Defense-Related Genes against Meloidogyne incognita Invasion in Tobacco
Source: Molecules. 2018 Aug 20;23(8):2081. doi: 10.3390/molecules23082081 (PMC6222693; doi:10.3390/molecules23082081)
Supplement: Supplementary file 1 [file molecules-23-02081-s001.zip › Table S1.pdf]

Table S1. Validated list of gene primers used for quantitative real time-PCR.

No.	Gene Symbol	Gene_ID	Forward primer	Reverse primer	Tm (°C)
1	PER47	gene_20009	GGGGGATTGTGAATGGTTAT	CCAAACAGACAACCAACATT	54
2	PER3	gene_9714	CTGGTATTATGTATCCTTGG	CCTCTCAATGTTAGATTAGG	50
3	ILR3	gene_7582	ACCAGGCTTTCTCTCCCATC	GCCACATCGCAACACTAGGA	59
4	RAP23	gene_13223	TAGCACCATTTTAGAGAGTT	AGTCATCCATCAGAAACATA	51
5	PR1	Novel00153	ATTGAACCATCCTGTAGTTA	CCTAATCTGGATAGAGTGTT	51
6	bgl13	gene_74391	CTGTCCGATTTGGCCTCACT	TATTGGACGGTGGTCCCTCT	59
7	PPO	gene_60360	TCGCCGAATAATGCTGAGTT	GTATAGCGGGAGCTTAGTGC	57
8	HMCS	gene_5079	GTTTGAAAGCAAGATCAGGG	GCTTGTTGTATGGTGAATGG	54
9	TGA	gene_2903	TGAAGCGTGGACTTTATTGA	TTGCCAATGAGCTTAATCCT	54
10	HST	gene_67788	CAGGTTTGGTATCGTTGTTG	GCCTCAACCCAATCTATCAA	54
11	SAMT	gene_78079	AACAAATTAAGGGTTCAATG	ACTACATAAATTAGCAGCTC	49
12	TPS9	gene_9996	GCTTTTCCCCCAATTTATAG	GCAAATTGTCAACCTTCTAA	51
13	FSPM	gene_85241	CTGCTACTGGTGAAGGTTAT	AGCACGGCTAACATTAAGAT	54
14	LAML	gene_82351	GTTTCTGTTGTAGTTCACCC	CATCAATCCAACCAACATCA	50
15	LEA5	gene_1320	GACTCTTAGCTCAATAACAA	TTGTTTGATTCCTCTACAC	54
16	L25	Internal reference	CCCCTCACCACAGAGTCTGC	AAGGGTGTTGTTGTCCTCAATCTT	56
