# Supplementary material for: Comparative Transcriptome Profiling Reveals Defense-Related Genes against Meloidogyne incognita Invasion in Tobacco
Source: Molecules. 2018 Aug 20;23(8):2081. doi: 10.3390/molecules23082081 (PMC6222693; doi:10.3390/molecules23082081)
Supplement: Supplementary file 1 [file molecules-23-02081-s001.zip › Table S2.pdf]

Table S2. Kyoto Encyclopedia of Genes and Genomes enriched pathways

Pathways	DEGs tested	All KEGG genes	*P*-value	Pathway ID
Phenylpropanoid biosynthesis	24	113	2.28E-15	ko00940
Tropane, piperidine and pyridine alkaloid biosynthesis	6	20	2.20E-05	ko00960
Tyrosine metabolism	5	23	0.000594648	ko00350
Isoquinoline alkaloid biosynthesis	4	14	0.000728791	ko00950
Pentose and glucuronate interconversions	6	39	0.001153767	ko00040
Starch and sucrose metabolism	7	102	0.03982093	ko00500
Monobactam biosynthesis	1	2	0.06261685	ko00261
Synthesis and degradation of ketone bodies	1	3	0.09246337	ko00072
Nitrogen metabolism	3	35	0.0975168	ko00910
Galactose metabolism	2	21	0.1421175	ko00052
Cyanoamino acid metabolism	2	21	0.1421175	ko00460
Phenylalanine metabolism	2	22	0.1532666	ko00360
Arginine biosynthesis	2	23	0.1645834	ko00220
beta-Alanine metabolism	2	24	0.1760465	ko00410
Alanine, aspartate and glutamate metabolism	3	50	0.2107882	ko00250
Glycine, serine and threonine metabolism	2	28	0.2229728	ko00260
Glycerophospholipid metabolism	2	28	0.2229728	ko00564
Sulfur metabolism	1	9	0.2528909	ko00920
Photosynthesis	1	9	0.2528909	ko00195
Selenocompound metabolism	1	9	0.2528909	ko00450
Nicotinate and nicotinamide metabolism	1	11	0.299897	ko00760
Butanoate metabolism	1	13	0.3439925	ko00650
Terpenoid backbone biosynthesis	1	16	0.4050636	ko00900
Biosynthesis of unsaturated fatty acids	1	18	0.4426356	ko01040
Circadian rhythm—plant	1	23	0.5266595	ko04712
alpha-Linolenic acid metabolism	1	23	0.5266595	ko00592
Fatty acid degradation	1	31	0.635902	ko00071

Valine, leucine and isoleucine degradation	1	32	0.6476793	ko00280
Arginine and proline metabolism	1	37	0.7011743	ko00330
Plant hormone signal transduction	3	119	0.7428598	ko04075
Plant-pathogen interaction	1	42	0.7466637	ko04626
Fatty acid metabolism	1	42	0.7466637	ko01212
Purine metabolism	1	46	0.7780904	ko00230
Carbon metabolism	2	90	0.7917009	ko01200
Glycolysis/Gluconeogenesis	1	53	0.8241258	ko00010
Phagosome	1	55	0.8354571	ko04145

DEGs: Differentially expressed genes
